# Frequency and correlates of malaria over-treatment in areas of differing malaria transmission: a cross-sectional study in rural Western Kenya

**DOI:** 10.1186/s12936-015-0613-7

**Published:** 2015-03-01

**Authors:** Frankline M Onchiri, Patricia B Pavlinac, Benson O Singa, Jacqueline M Naulikha, Elizabeth A Odundo, Carey Farquhar, Barbra A Richardson, Grace John-Stewart, Judd L Walson

**Affiliations:** Department of Epidemiology, University of Washington, 325 Ninth Avenue, Box 359909, Seattle, WA 98104 USA; Kenya Medical Research Institute, Centre for Clinical Research, Nairobi, Kenya; Department of Pediatrics, University of Washington, Seattle, USA; Department of Biostatistics, University of Washington, Seattle, USA; Department of Global Health, University of Washington, Seattle, USA; Department of Medicine, University of Washington, Seattle, USA; Vaccine and Infectious Disease Division, Fred Hutchinson Cancer Research Center, Seattle, USA; Walter Reed Project, United States Army Medical Research Unit, Kericho, Kenya

**Keywords:** Malaria, Laboratory testing, Over-treatment

## Abstract

**Background:**

In 2010, the World Health Organization shifted its malaria guidelines from recommending the empiric treatment of all febrile children to treating only those with laboratory-confirmed malaria. This study evaluated the frequency and predictors of malaria over-treatment among febrile malaria-negative children in Kenya.

**Methods:**

Between 2012 and 2013, 1,362 children presenting consecutively with temperature ≥37.5°C to Kisii and Homa Bay hospitals were enrolled in a cross-sectional study evaluating causes of fever. Children were screened for malaria using smear microscopy and rapid diagnostic tests and managed according to standard of care at the hospitals. The frequency of anti-malarial prescriptions among children with laboratory-confirmed malaria negative children (malaria over-treatment) was determined; and clinical and demographic correlates of overtreatment evaluated using logistic regression. Because of differences in malaria endemicity, analyses were stratified and compared by site.

**Results:**

Among 1,362 children enrolled, 46 (7%) of 685 children in Kisii, and 310 (45.8%) of 677 in Homa Bay had laboratory-confirmed malaria; p < 0.001. Among malaria-negative children; 210 (57.2%) in Homa Bay and 45 (7.0%) in Kisii received anti-malarials; p < 0.001. Predictors of over-treatment in Homa Bay included ≥ one integrated management of childhood illness (IMCI) danger sign (aOR = 8.47; 95% CI: 4.81-14.89), fever lasting ≥ seven days (aOR = 4.94; 95% CI: 1.90-12.86), and fever ≥39°C (aOR = 3.07; 95% CI: 1.58-5.96). In Kisii, only fever ≥39°C predicted over-treatment (aOR = 2.13; 95% CI: 1.02-4.45).

**Conclusions:**

Malaria over-treatment was common, particularly in Homa Bay, where the prevalence of malaria was extremely high. Severe illness and high or prolonged fever were associated with overtreatment. Overtreatment may result in failure to treat other serious causes of fever, drug resistance, and unnecessarily treatment costs.

## Background

Malaria is a leading cause of morbidity and mortality among children in sub-Saharan Africa. The most well recognized sign of malaria infection is fever, which accounts for over 30-50% of all paediatric hospital visits in sub-Saharan Africa [1]. Historically, international guidelines recommended presumptive treatment of malaria in febrile children in endemic areas, as diagnostic tools were infrequently available and cost prohibitive and malaria-attributable mortality that would occur if treatment was delayed was high [2]. Following considerable declines in malarial transmission in many parts of Africa, including in highly endemic areas [3-5], the increased recognition of the importance of life-threatening non-malaria fevers [6-8] and the wide availability of cheap and highly sensitive rapid diagnostic tests (RDTs), the World Health Organization released new malaria treatment guidelines in 2010 that recommend limiting treatment to laboratory-confirmed malaria [9]. While many countries, including Kenya, have adopted these guidelines with subsequent improvements in malaria case-management practices [10], a considerable proportion of febrile patients still continue to be treated for malaria despite negative laboratory test (over-treatment) [10-12].

Malaria overtreatment may result in the failure to treat other serious causes of fever, particularly blood stream infections [7,8]. Mortality among children treated for malaria is over two-fold higher in malaria-negative children than in children with laboratory-confirmed malaria, often as a result of untreated bacterial infections [7,8,13]. Additionally, malaria over-treatment can lead to the emergence of drug resistance, unnecessary adverse drug effects, increased treatment costs, and reduced quality of care [14,15]. Because of the serious potential consequences of malaria over-treatment, it is important to understand why some children are inappropriately treated with anti-malarials. This information will inform fever management and may improve understanding of guideline adherence in low-resource settings.

## Methods

### Study design, sites, and population

This was a cross-sectional study nested within an ongoing surveillance study of febrile illnesses among children at Kisii Provincial Hospital and Homa Bay District Hospital, both in Western Kenya. These hospitals are situated in areas with historically different malaria endemicity: Kisii is hypo-endemic with annual entomologic inoculation rate (EIR) of <1.5 infectious bites per person per year. Homa Bay historically has been holo-endemic with EIR of ≥300 infectious bites per person per year [16,17], although malaria prevalence declined by over 50% from 2003 to 2007 [4,5] but then reversed to levels observed in 2003 [18]. Children ages six months to 15 years presenting with axillary temperature ≥37.5°C at the out-patient departments (OPD) were approached consecutively for enrolment and almost all were enrolled in OPD (98%). A minority of study children (<2%) were admitted to the wards directly from OPD and enrolled from the inpatient ward. Eligible children included those whose accompanying parents/guardians consented to HIV counseling and study participation of their children. Because this study was nested within a large surveillance study of febrile illnesses, data from all children enrolled at the time (April 2012 – November 2013) of analysis presented in this paper were used. The study was approved by the Ethical Review Committees of both the University of Washington and the Kenya Medical Research Institute.

### Study and hospital clinical staff

Clinical staff at the study sites consists of clinical officers and registered nurses with three years of medical training and licensed to independently diagnose and treat diseases, including ordering and interpreting laboratory tests. There are very few medical doctors available for direct clinical care. The new malaria “test and treat” policy was adopted by the Kenyan Ministry of Health in 2011, and subsequently health workers received in-service malaria case-management training [11]. However, structured monitoring and supervision of malaria treatment and treatment practices are not routinely available. Study clinicians/nurses worked together with hospitals’ clinical staff and assisted with the diagnosis and treatment of patients regardless of their eventual study participation statues.

### Training

Study staff was trained on enrolment criteria, consent, clinical and laboratory procedures, use of integrated management of childhood illness (IMCI) syndromic management algorithms, and assessment of danger signs. Because the parent study was a cross-sectional study aimed at understanding bacterial etiologies of fever, the study protocol did not stipulate standardized treatment protocols for malaria and clinicians managed sick children according to the standard of care practices at the hospitals.

### Data collection

During screening and enrolment, study clinicians and registered nurses systematically captured comprehensive information regarding sociodemographics, anthropometric characteristics, past medical history and care-seeking behaviour, and current clinical and laboratory data using standardized case report forms. Clinicians also recorded diagnoses and prescribed treatments. Children with any of the IMCI danger signs (unable to drink or breastfeed, convulsions, continuous vomiting, lethargy, and/or unconsciousness) were classified as having severe febrile illness [19]. All screened and enrolled children were tested for malaria using both smear microscopy and Paracheck Pf® RDTs (Orchid Biomedical Services, India). At each hospital, the same hospital microscopists prepared and read malaria slides. Malaria results were available to clinicians within 20–30 minutes of testing. These microscopists received no additional training before the study. Because causes of fever such as bacteraemia are associated with HIV [20] and malaria [21], children were tested for HIV using rapid antibody testing (Abbott Determine™ rapid test kit and confirmed using Uni-Gold™) or PCR if child ≤18 months. Finally, blood was cultured using BACTEC 9050 blood culture system, and pathogens were identified using a Microscan Walk away system. Results of the analyses of bacteremia data are presented elsewhere [22].

### Study outcomes

Laboratory-confirmed malaria was defined as a positive smear microscopy and/or RDT test. Malaria over-treatment was defined as a negative smear and RDT result that was treated with anti-malarials.

### Statistical analysis

Demographic, clinical and laboratory characteristics were compared between study sites using Chi-square or Fisher’s exact tests for categorical variables and two sample t-tests or Wilcoxon rank sum tests for continuous variables.

The proportion of children laboratory-confirmed negative malaria but were treated with anti-malarials was estimated and compared between sites using Chi-square test. The association between malaria over-treatment (primary outcome) and patients’ clinical and demographic characteristics that had been selected *a priori* following a thorough literature review was first examined in a bivariate logistic regression. These included malnutrition, mother, and child’s HIV status, presence of IMCI danger signs, fever of ≥ seven days, having previously sought care, pre-treatment with anti-malarial prior to hospitalization. To identify independent correlates of malaria over-treatment, all of the following *a priori* selected confounders were included simultaneously in a multivariate logistic model: child’s age, sex, whether the child was enrolled from out- or in-patient departments, primary caregiver’s level of education and income. The latter two variables were used as surrogates for family socioeconomic status. Because the sites had marked differences in malaria endemicities that might impact clinician suspicion, analyses were stratified and compared by site. All analyses were done using Stata statistical software (version 13.1, Stata Corp, College Station, TX, USA). Strengthening the reporting of observational studies in epidemiology (STROBE) guidelines were followed in reporting this study [23].

## Results

### Demographic, medical history and clinical characteristics

**Figure 1:**
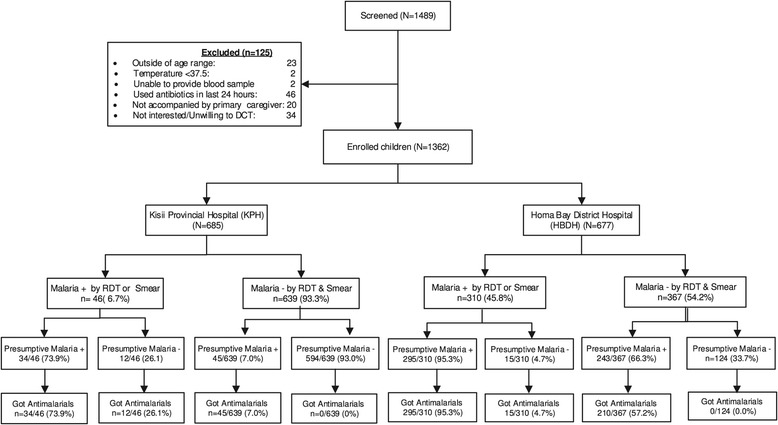
**Flow chart showing enrolment of children by study site, laboratory, clinical diagnosis of malaria and treatment.**

Between April 2012 and November 2013, 1,362 febrile children were enrolled into the parent study; 685 in Kisii and 677 in Homa Bay. Figure 1 summarizes screening, enrolment, clinical treatment of malaria, and laboratory testing for malaria. Baseline demographic and clinical information of the enrolled children are shown in Table 1. Compared to children from Kisii, children from Homa Bay were younger, more likely to be female, more likely to be acutely malnourished (underweight), not to have received all age-appropriate vaccines, and sicker at presentation as defined by the presence of IMCI danger sign(s) (52.6% *vs* 16.1%; p < 0.001). Children in Homa Bay were more likely to have HIV-infected mothers (19.5% *vs* 3.3%; p < 0.001) or to be HIV infected themselves (4.5% *vs* 1.3%; p < 0.001) than those from Kisii. However, children from Homa Bay were less likely to be wasted, and to have previously sought health care elsewhere for their current illness than children from Kisii (23.0% *vs* 33.9%; p = 0.001).

**Table 1:** **Demographic and clinical characteristics of children by site**

		**Study site**		
**Variable**	**All sites**	**Kisii**	**Homa Bay**	
	**n %**	**n (%)**	**n (%)**	**p**
	**n = 1,362**	**n = 685**	**n = 677**	
**Demographics**				
Child’s sex: Female	652 (44.7)	306 (44.7)	346 (51.1)	0.017
Age in years (Mean ± SD) (Median)	35.4 ± 34 (34)	3.1 ± 2.1 (2.9)	2.8 ± 1.6 (2.8)	0.012
Age (years)				<0.001
≤2 yr	501 (36.8)	256 (37.4)	245 (36.2)	
3-4 yr	721 (53.0)	335 (49.0)	386 (57.1)	
≥5 yr	138 (10.1)	93 (13.6)	45 (6.7)	
Uses water from unimproved sources^i^	171 (12.6)	26 (3.8)	145 (21.4)	<0.001
**Primary caregiver’s characteristics**				
Education				<0.001
College/university	159 (11.7)	101 (14.8)	58 (8.6)	
Secondary	425 (31.2)	273 (39.9)	152 (22.5)	
At most primary	777 (57.1)	310 (45.3)	467 (69.0)	
Monthly income				<0.001
≤$50	550 (40.4)	189 (27.6)	361 (53.3)	
$50-100	495 (36.4)	275 (40.2)	220 (32.5)	
>$100	316 (23.2)	220 (32.2)	96 (14.2)	
**Children’s clinical presentations**				
Malnutrition				
MUAC < 12.5	40 (2.9)	15 (2.2)	25 (3.7)	0.087
Wasting (WHZ < −2)^ii^	193 (15.9)	115 (19.5)	78 (12.4)	0.001
Stunted (HAZ < −2)^ii^	181 (14.5)	83 (13.6)	98 (15.4)	0.272
Underweight (WAZ < −2)^iii^	127 (10.2)	52 (8.5)	75 (11.8)	0.044
Temperature (°C)				<0.001
37.5-37.9	205 (15.1)	138 (20.1)	67 (9.9)	
38.0-38.9	626 (46.0)	308 (45.0)	318 (47.0)	
39.0-39.9	441 (32.4)	202 (29.5)	239 (35.3)	
> = 40.0	90 (6.6)	37 (5.4)	53 (7.8)	
High grade fever (>39°C)	468 (34.4)	206 (30.1)	262 (38.7)	0.001
≥IMCI danger sign(s) present^iv^	466 (34.2)	110 (16.1)	356 (52.6)	<0.001
Unable to drink/breastfeed	198	14	184	
Vomits everything	271	62	209	
Convulsions	81	30	51	
Lethargic/unconscious	113	43	70	
**Past clinical history**				
Has had fever lasting ≥7 days		263 (38.4)	79 (11.8)	<0.001
Sought healthcare elsewhere	388 (28.5)	232 (33.9)	156 (23.0)	<0.001
Taken anti-malarials last 7 days	192 (14.1)	86 (12.6)	106 (15.7)	0.1000
Taken antibiotics last 7 days	84 (6.2)	52 (7.6)	32 (4.7)	0.024
Not received all age-appropriate vaccines	324 (23.8)	11 (11.8)	243 (35.9%)	<0.001
**Laboratory results**				
Mother HIV -infected^v^	151 (11.4)	22 (3.3)	129 (19.5)	<0.001
Child HIV-infected^vi^	39 (2.9)	9 (1.3)	30 (4.5)	0.001
Malaria (smear or RDT)	356 (26.1)	46 (6.7)	310 (45.8)	<0.001
Bacteraemia	52 (3.8)	27 (3.9)	25 (3.7)	0.811

^i^Unpiped water, unprotected boreholes, wells and springs, water from vendors.

^ii^Values outside plausible range for 95 children from Kisii and 50 from Homa Bay.

^iii^Values outside plausible range for 74 children from Kisii and 42 from Homa Bay.

^iv^Unable to feed/drink, vomits everything, convulsion, unconscious, stiff neck.

^v^Mother’s HIV status unknown for 25 children from Kisii and 15 from Homa bay.

^vi^HIV status uknown for 15 children from Kisii and 3 from Homa Bay.

### Malaria diagnoses

Among febrile children, 26% had laboratory-confirmed malaria infection; 45.8% in Homa Bay and 6.7% in Kisii; prevalence ratio = 6.82; 95% CI: 5.22-8.90. Among laboratory-confirmed malaria cases (n = 356), 96.1% were positive by both RDT and smear, 10 (2.8%) were positive by RDT alone and 4 (1.1%) were positive by smear alone. In Kisii, the prevalence of bacteraemia among children with and without malaria was 4.1% (26/639) and 2.2% (1/46), respectively, p = 0.524. The prevalence of bacteremia among children with and without malaria was 4.5% (45/1006) and 2.0% (7/35), respectively; p = 0.034.

### Malaria treatment and correlates of malaria over-treatment

All children with laboratory-confirmed malaria (n = 356) were treated with anti-malarials regardless of their initial provisional presumptive diagnosis. Most (82.3% received artemisinin combination therapy (ACT), while 17.7% received quinine. Children treated with quinine were more likely than those treated with ACT to have danger sign(s) present 76.2% (48/63) vs. 47.1% (138/293); p < 0.001. Among children with laboratory-confirmed negative malaria (639 in Kisii and 367 in Homa Bay), the proportion of those treated with anti-malarials was significantly higher in Homa Bay as compared to Kisii (57.2% vs. 7.0% p < 0.001). Overall, among those over-treated, children treated with quinine were more likely than those treated with ACT to have danger sign(s) present 76.2% (48/63) vs. 47.1% (138/293); p < 0.001.

Because the site characteristics and patients’ demographic and clinical characteristics were so distinct, analyses were stratified and compared by site. In Kisii, odds of malaria over-treatment were significantly higher for children with high-grade fever (odds ratio (OR) = 2.14; 95% CI: 1.21-4.37), those who had taken anti-malarials in the week preceding hospitalization (OR = 2.14; 95% CI: 1.03-4.44) and those whose mothers were HIV-infected (OR = 3.26; 95% CI: 1.11-9.58) in bivariate analysis (Table 2). In multivariate analysis, only high-grade fever was associated with over-treatment (aOR = 2.13; 95% CI: 1.02-4.45).

**Table 2:** **Univariate and multivariate analysis of correlates of malaria over-treatment in Kisii**

	**Over-treated**		**Crude**	**Adjusted** ^§^
	**No (n = 594)**	**Yes (n = 45)**	**cOR (95% CI)**	**aOR (95% CI)**
**Potential correlates**	**n (%)**	**n (%)**		
Malnutrition				
MUAC < 12.5	13 (2.2)	1 (2.2)	1.02(0.13-7.94)	1.57(0.18-13.42)
Wasting	107 (20.8)	5 (14.3)	0.64(0.24-1.68)	0.70(0.20-1.89)
Stunted	73 (13.7)	3 (8.6)	0.59(0.18-1.98)	0.75(0.18-3.01)
Underweight	45 (8.4)	2 (5.7)	0.66(0.15-2.83)	1.12(0.18-7.12)
Mother HIV-infected	17 (3.0)	4 (9.1)	3.26(1.11-9.58)	1.81(0.39-8.43)
Child HIV-infected	7 (1.2)	2 (4.5)	3.90(0.89-17.30)	NA^†^
Any IMCI danger sign?	90 (15.2)	8 (17.8)	1.21(0.55-2.69)	1.94(0.73-5.17)
High grade fever (>39°C)	166 (27.9)	21 (46.7)	2.26(1.24-4.16)	2.13(1.02 - 4.45)
Fever for 7 or more days	232 (39.1)	19 (42.2)	1.14(0.62-2.11)	1.48(0.68-3.21)
Sought care elsewhere	195 (32.8)	14 (31.1)	0.92(0.48-1.78)	0.83(0.33-2.06)
Taken anti-malarial last 7 days	70 (11.8)	10 (22.2)	2.14(1.03-4.44)	2.45(0.84-7.29)
Taken antibiotics last 7 days	47 (7.9)	2 (4.4)	0.54(0.13-2.30)	0.31(0.04-2.84)

^†^Data insufficient to include this variable in multivariate model only two of the children over-diagnosed in Kisii were HIV+. ^§^Adjusted for child’s age, sex, and caregivers’ education and household income.

In Homa Bay, signs and symptoms of severe sickness were associated with over-treatment. The unadjusted odds of malaria over-treatment for children with both a negative blood smear and RTD test result were significantly higher for children with high-grade fever (OR = 2.07; 95% CI: 1.27-3.39), a history of fever lasting a week or more (OR = 2.94; 95% CI: 1.41-6.14), and at least one IMCI danger signs present (OR = 5.78; 95% CI: 3.66-9.14) (Table 3). However, the odds of over-treatment were lower for children who reported seeking care prior to current hospital visit (OR = 0.49; 95% CI: 0.30-0.79) and lower for children who had reported having taken antibiotics within the last one week (OR = 0.42; 95% CI: 0.18-0.98). These associations persisted and strengthened in multivariate logistic regression analyses adjusting simultaneously for *a priori* selected confounders that included sex, age of the children, whether children were enrolled from in-patient or outpatient department, and primary caregivers’ education and income. In multivariate analysis, malaria over-treatment in Homa Bay was significantly associated with high-grade fever ≥39°C (aOR = 3.07; 95% CI: 1.58-5.96), a history of fever lasting a week or more (aOR = 4.98; 95% CI: 1.90-12.80), and presence of at least one IMCI danger sign (aOR = 8.47; 95% CI: 4.81-14.89). Notably, there was no significant association of malaria over-treatment with HIV-infection.

**Table 3:** **Univariate and multivariate analysis of correlates of malaria over-treatment in Homa Bay**

	**Over-treated**		**Crude**	**Adjusted** ^§^
**Variable**	**No (n = 157)**	**Yes (n = 210)**	**cOR (95% CI)**	**aOR (95% CI)**
**Potential correlates**	**n (%)**	**n (%)**		
Malnutrition				
MUAC < 12.5	4 (2.5)	10 (4.8)	1.91(0.60-6.21)	1.73(0.37-8.05)
Wasting	17 (11.4)	26 (13.6)	1.22(0.64-2.35)	0.67(0.25-1.77)
Stunted	19 (12.5)	33 (17.1)	1.44(0.78-2.68)	1.24(0.53-2.89)
Underweight	14 (9.2)	27 (14.0)	1.60(0.81-3.18)	0.93(0.30-2.89)
Mother HIV-infected	30 (19.2)	45 (22.0)	1.18(0.70-2.00)	1.19(0.59-2.50)
Child HIV-infected	6 (3.9)	15 (7.1)	1.90(0.72-5.01)	1.36(0.35-5.27)
Any IMCI danger sign?	41 (26.1)	141 (67.1)	5.78(3.66-9.14)	8.47(4.81-14.89)
High grade fever (>39°C)	30 (19.1)	69 (32.9)	2.07(1.27-3.39)	3.07(1.58-5.96)
Fever for 7 or more days	10 (6.4)	35 (16.7)	2.94(1.41-6.14)	4.94(1.90-12.86)
Sought care elsewhere	48 (30.6)	37 (17.6)	0.49(0.30-0.79)	0.32(0.18-0.63)
Taken anti-malarial last 7 days	26 (16.6)	50 (23.8)	1.57(0.93-2.67)	1.95(0.95-4.00)
Taken antibiotics last 7 days	15 (9.6)	9 (4.3)	0.42(0.18-.98)	0.17(0.05-0.55)

^§^Adjusted for child’s age, sex, and caregivers’ education and household income.

### Antibiotic treatment

More children with negative laboratory malaria diagnostic tests were treated with both anti-malarials and antibiotics in Homa Bay than in Kisii (45.8 *vs* 6.3%; p < 0.001). In Homa Bay, children more likely to be treated with both anti-malarials and antibiotics included those who presented with a history of fever lasting a week or more (OR = 2.38; 95% CI: 1.09-5.20) or manifested an IMCI danger sign(s) (OR = 4.58; 95% CI: 2.78-7.22). In Kisii, although children presenting with high-grade fever of ≥39°C but without malaria were more likely to receive both anti-malarials and antibiotics (OR = 2.26; 95% CI: 1.22-4.16), the presence of IMCI danger signs was not predictive of dual treatment (OR = 1.21; 95% CI: 0.55-2.69).

## Discussion

Current malaria treatment guidelines recommend restricting anti-malarials to confirmed malaria cases only. This study assessed anti-malarial prescribing practices at two regional rural hospitals in western Kenya and found that malaria endemicity appears to be a major driver of clinicians’ adherence to malaria laboratory test results.

Clinicians correctly adhered to positive test results and prescribed all malaria-positive children with anti-malarials at both sites. However, more than half (57.2%) of febrile children with laboratory-confirmed negative malaria in a high malaria transmission area were prescribed anti-malarials compared to only 7.0% in a low malaria transmission area. In Kisii, the only strong predictor of malaria over-treatment was a fever of >39°C. In Homa Bay, signs of more severe illness (high-grade fever >39°C, longer duration of fever, IMCI danger signs) were the strongest predictors of over-treatment with anti-malarials. In high malaria prevalence areas, clinicians may have over-treated sick children because they did not trust negative test results, and/or feared missing possible malaria cases, with potentially fatal consequences [24]. The finding that overtreatment is more common in an area of high malaria transmission is consistent with what might be expected: when prevalence of any disease is relatively high, a clinician’s suspicion of the disease prior to testing is also high to avoid missing causes of disease that are common in a given area.

The higher proportion of children with danger signs in Homa Bay may, in part, reflect limited healthcare exposure, delayed access to, or poor engagement with, the health care system and inappropriate home treatment of children with fever. Additionally, significantly fewer children in Homa Bay (the high malaria risk area) had received all age-appropriate vaccines and children in Homa Bay were also significantly more likely to be HIV-infected or exposed. Because study procedures, inclusion criteria, and training of study clinicians were uniform across study sites, it is unlikely that the higher proportion of very sick children in Homa Bay was due to systematic selection bias or differential misclassification.

This study found that children exhibiting signs of severe disease were more likely to be treated for both malaria and for presumptive blood stream infection. More than 80% of the children at both sites were presumptively prescribed antibiotics. This suggests that in the absence of point-of-care diagnostics for other causes of fever, clinicians are reluctant to miss the opportunity to avert potential morbidity and mortality and will presumptively treat children, even when guidelines suggest otherwise. Malaria-negative children were more likely to be prescribed antibiotics compared to children with positive results (93.3 vs. 58.2%, p < 0.001), suggesting that negative malaria test may have led clinicians to consider and treat alternative causes of fever.

The finding of significant malaria over-treatment, particularly in very sick children in endemic areas, is consistent with a recent Cochrane review of the effect of RDTs on the prescription of anti-malarials in Africa [24]. This review reported that underlying malaria prevalence influences clinicians’ prescribing adherence to malaria test results. Findings from this study have important practical implications for regular refresher in-service trainings on importance of parasitological-supported malaria treatment, and structured supervision of clinicians to increase their adherence to “test and treat” policy as well as their confidence in malaria test results. Given the concern of missing possible malaria infections, training should reiterate findings from multiple trials which have shown that although RDTs may miss some uncomplicated malaria infections, these are mainly low-density and self-limiting infections, which rarely develop into severe malaria [25]. The finding that very sick-looking children were treated with anti-malarials despite negative laboratory tests suggests the need to educate clinicians that withholding anti-malarials from febrile malaria-negative patients is clinically safe, even in endemic areas [24-27]. Because malaria tests, particularly RDTs, may be the only diagnostic tools routinely available in rural areas in Kenya, it is necessary to train, support, and supervise clinicians on the use of test result as basis for prescribing anti-malarials, and that a negative test should prompt an assessment and treatment of the true causes of fever.

While this study had several strengths, notably the inclusion of data from two sites in Kenya with widely differing underlying malaria endemicity, there were also some weaknesses. Malaria over-treatment was only evaluated in relation to patients’ demographic and their clinical symptoms. Other factors such as clinicians’ level of training, number of clinical staff and patient load, patients’ expectations, diagnostic laboratory capacity, and regular supervision and public health promotional activities also likely affect clinicians’ malaria diagnosis and treatment decisions. Lastly, although the inclusion of only children with fever ≥37.5°C was intended to enhance the clinical yield of microbiologic investigations, this may limit the generalizability.

## Conclusions

Febrile patients, particularly those with signs of more severe illness in areas of high malaria transmission, continue to be treated with anti-malarials despite negative laboratory tests, against current WHO “test-treat” policy. This may result in missed opportunities to accurately diagnose and treat alternative causes of fever. There is need for the strengthened training in fever case management, supervision, monitoring and evaluation of malaria treatment practices by clinicians in order to ensure adherence to malaria test results, particularly in areas of high malaria endemicity.
